# Single-Cell Analysis Reveals STARD10 as a Fatty Acid Metabolism Regulator of Breast Cancer Progression via the PI3K/Akt Pathway

**DOI:** 10.3390/ijms27167237

**Published:** 2026-08-13

**Authors:** Yining Han, Jiacheng Fan, Yue Xi, Pengxiang Zhu, Qiyue Sun, Xiaofeng Li

**Affiliations:** Department of Epidemiology and Health Statistics, Dalian Medical University, Dalian 116044, China; hanyn01@dmu.edu.cn (Y.H.);

**Keywords:** single-cell RNA sequencing, breast cancer, fatty acid metabolism, PI3K/Akt, STARD10

## Abstract

Breast cancer is a highly heterogeneous malignancy in which fatty acid metabolism plays a critical yet insufficiently characterized role in tumor progression, immune evasion, and treatment resistance. To address this gap, we performed a comprehensive re-analysis of single-cell transcriptomic data from 62 breast cancer patients using the Non-negative Matrix Factorization (NMF) algorithm to delineate tumor cell subpopulations at single-cell resolution. Through Gene Set Enrichment Analysis (GSEA), we identified the tumor cell subgroup most significantly associated with fatty acid metabolism. Subsequent Slingshot trajectory analysis revealed STARD10 as a key regulatory gene in fatty acid metabolic reprogramming along tumor cell differentiation trajectories. In vitro functional experiments further validated that STARD10 plays a functional role in modulating fatty acid metabolism in breast cancer cells. These findings establish STARD10 as a novel molecular marker and potential therapeutic target in breast cancer, offering new insights into the metabolic mechanisms underlying tumor heterogeneity and disease progression.

## 1. Introduction

Breast cancer is still one of the most common malignant tumors among women around the world. According to the statistics of GLOBOCAN 2022, there were about 2.3 million new cases of breast cancer in the world that year, accounting for 11.6% of all new cancers [1]. In China, there were about 357,200 new cases of breast cancer and about 75,000 deaths in 2022, accounting for 15.59% and 7.94% of female cancer cases and deaths, respectively [2], indicating that the disease still puts a heavy burden on public health. It should be pointed out that breast cancer cannot be simply regarded as a single clinical pathological type, but a class of diseases with significant biological heterogeneity. There are obvious differences at many levels, including histopathological findings, molecular typing, genomic characteristics, transcriptional status, and the tumor microenvironment. Whether it occurs between tumors or within tumors, it deeply affects tumor biological behavior and treatment response and is closely related to recurrence, metastasis, and acquired drug resistance [3,4,5,6]. Therefore, it is of great value to optimize hierarchical patient management and to promote precise treatment by deeply elucidating the molecular and cytological mechanisms underlying breast cancer heterogeneity and by screening for important markers related to tumor progression and treatment responses.

Although traditional bulk RNA sequencing can capture the gene expression spectrum at the tissue level, it essentially reflects the average signal after mixing different cell groups. Therefore, this method is difficult to accurately distinguish the respective molecular characteristics of tumor cells, immune cells, and matrix cells, and it is not enough to analyze key biological processes such as rare cell subgroups, cell state transformation, and treatment-related clonal evolution [7,8]. In recent years, single-cytomic technology has developed rapidly, which has significantly improved the resolution of tumor research. Among them, single-cell RNA sequencing (scRNA-seq) can systematically portray the dynamic changes in cell heterogeneity, spectrum evolution, and functional state at the single-cell level, which provides a powerful means to clarify the mechanism of breast cancer occurrence and development [8,9]. Studies have shown that scRNA-seq can not only analyze the state heterogeneity of breast cancer epithelial cells in more detail, but also simultaneously reveal the composition and interaction of immune cells and stromal cells in the tumor microenvironment, thereby providing higher-resolution evidence for understanding tumor progression, immune evasion, and differential treatment responses [9,10].

Metabolic reprogramming has been widely regarded as one of the important biological characteristics of tumors, and the role of lipid metabolism, especially fatty acid metabolism, in the biology of breast cancer is also increasingly valued. More and more studies show that fatty acid metabolism is not only involved in basic processes such as membrane lipid synthesis, energy storage, and oxidation supply, but also closely related to a variety of malignant biological behaviors such as proliferation, invasion, metastasis, immune escape, and treatment tolerance of tumor cells. Within the adipose-rich tumor milieu of breast cancer, dysregulated fatty acid metabolism may further influence disease progression and therapeutic sensitivity through remodeling of the tumor immune microenvironment [10,11]. Notably, single-cell studies have directly demonstrated that enhanced fatty acid metabolism is associated with metastatic potential and a low-immune-infiltration phenotype in breast cancer, accompanied by metabolic reprogramming of immune cells within the tumor microenvironment [12,13,14]. While the synthesis, uptake, and storage of fatty acids in breast cancer have been increasingly characterized, the intracellular transport of lipids—particularly the trafficking of fatty acids and other lipid species between membranes and organelles—remains comparatively understudied. This process is largely governed by lipid transfer proteins, among which the steroidogenic acute regulatory protein (StAR)-related lipid transfer (START) domain family plays a central role in mediating non-vesicular lipid transport between intracellular compartments [15]. STARD10, a member of this family with selective binding for phosphatidylcholine and phosphatidylethanolamine, has been reported to be overexpressed in both murine and human breast tumors and to cooperate with c-erbB signaling in ERBB2-positive breast cancer, where it is highly expressed in 35–40% of cases and promotes malignant progression [16]. Notably, loss of STARD10 expression has been independently associated with poor disease-specific survival in breast cancer patients, even after adjusting for tumor size, grade, lymph node status, hormone receptor, and HER2 status [17], suggesting that STARD10 may exert context-dependent, dual roles in breast cancer depending on molecular subtype and signaling background. Despite these clinical associations, the mechanisms by which STARD10-mediated lipid transport intersects with fatty acid metabolic reprogramming in breast cancer, and how this relationship varies across distinct tumor cell populations, remain poorly defined.

## 2. Results

### 2.1. Establishment of Single-Cell Landscape of BRCA and Normal Breast Tissue

**Figure 1:**
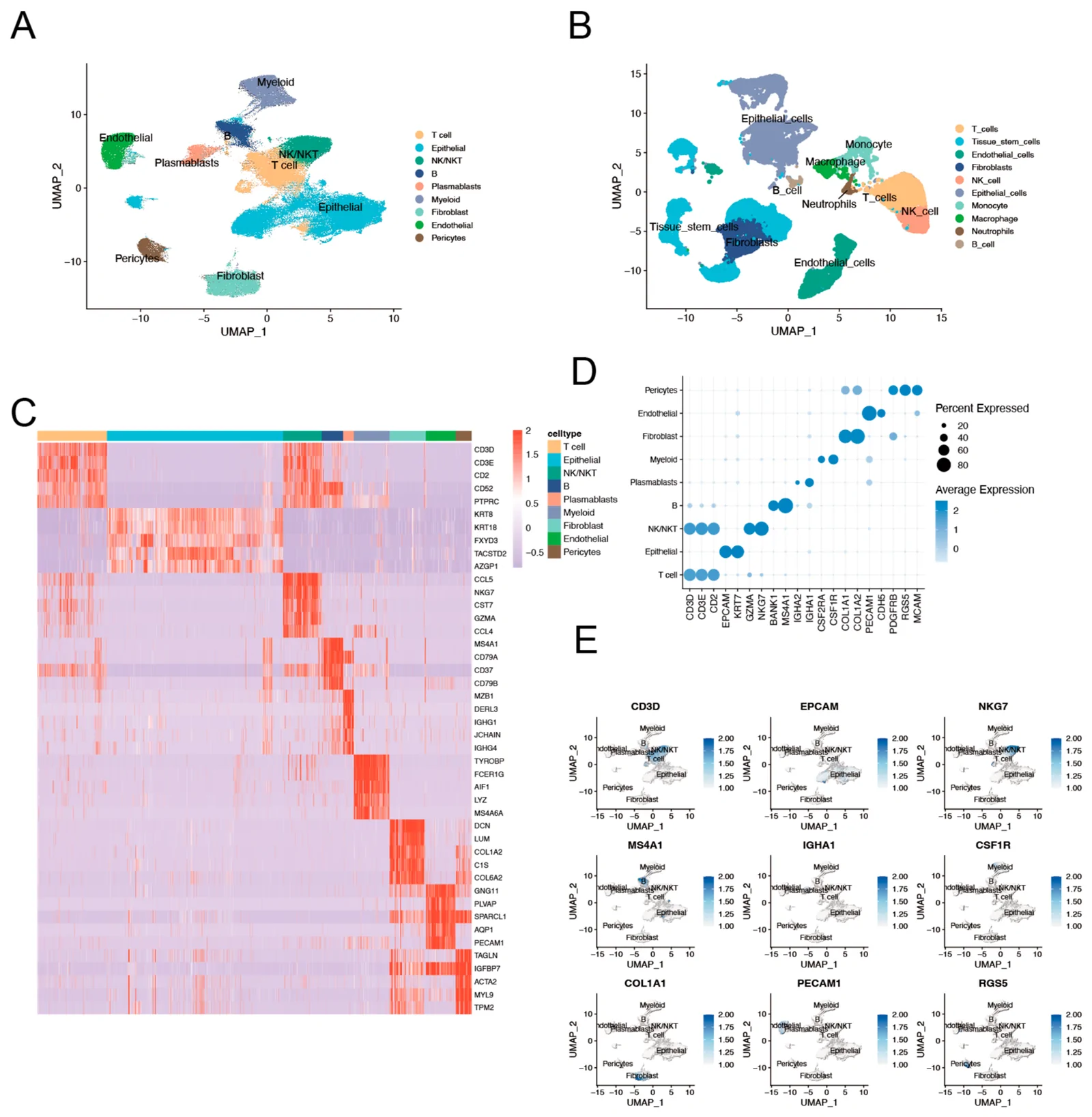
Single-cell transcriptomic landscape of breast cancer patients (*n* = 194,067 cells from 62 samples). (**A**) UMAP visualization of annotated cell subpopulations in breast cancer samples. (**B**) Cell subpopulation annotation of normal breast samples based on the SingleR algorithm (*n* = 6 samples from GSE161529) based on the SingleR algorithm. (**C**) Heatmap of differentially expressed genes across the identified cell types (FindAllMarkers function in Seurat; adjusted *p* < 0.05, |log2FC| > 0.25). (**D**) Bubble plot showing key marker genes for each cell subpopulation. (**E**) UMAP feature plots illustrating the expression and distribution of key marker genes across cell subpopulations.

**Figure 2:**
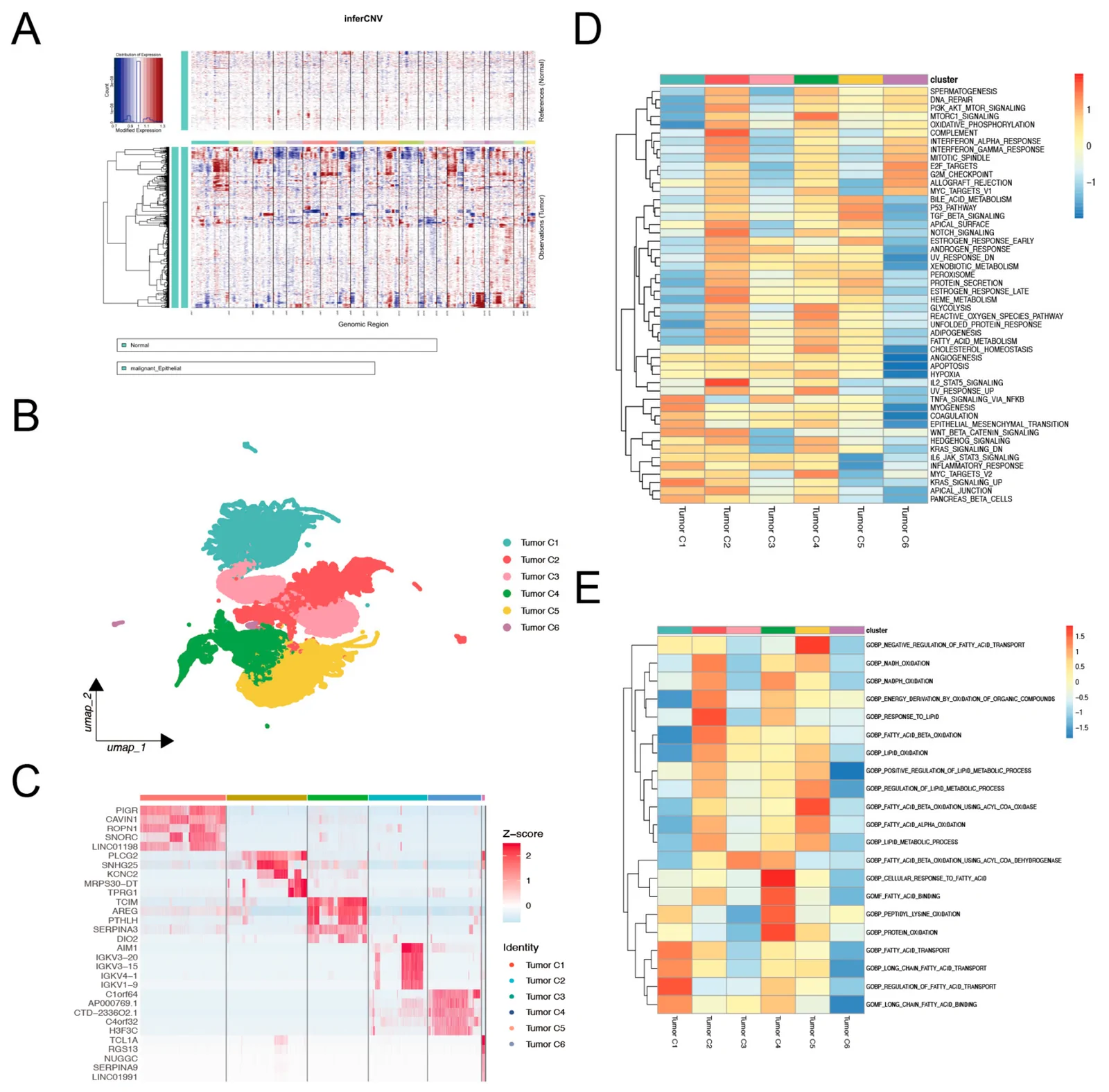
Identification of fatty acid metabolism-related subpopulations in breast cancer. (**A**) Inferred copy number variation profiles based on inferCNV. (**B**) UMAP plot showing six clustered tumor cell subpopulations (Tumor C1–Tumor C6). (**C**) Heatmap showing the marker genes of each tumor cell cluster (FindAllMarkers function in Seurat; adjusted *p* < 0.05, |log2FC| > 0.25). (**D**) Heatmap of Hallmark pathway enrichment analysis based on gene sets from the MSigDB database (h.all.v2024.1.Hs.symbols). (**E**) Heatmap of Gene Ontology (GO) enrichment analysis. Created in Adobe Illustrator 2023 (version 27.0).

Single-cell RNA sequencing data were obtained from the Gene Expression Omnibus (GEO). In this study, three scRNA-seq datasets, GSE176078, GSE180878, and GSE195861, comprising a total of 62 samples, were integrated for subsequent analysis. Batch effects across the 62 samples were corrected using the Harmony package, followed by single-cell quality control. After removal of low-quality cells, a total of 194,067 cells were retained for downstream analysis. Using a clustering resolution of 0.6 and 50 principal components (PCs), cell subpopulations were annotated on the basis of established marker genes curated from the CellMarker database, resulting in the identification of 13 major cell types (Figure 1A). These included epithelial (tumor) cells, NK/NKT cells, proliferating T cells, regulatory T cells (Treg), follicular helper T cells (Tfh), naive T cells, central memory T cells (TCM), B cells, plasma cells, myeloid cells, fibroblasts, endothelial cells, and pericytes. To validate the annotation results, heatmaps displaying the top five canonical marker genes for each cell type were generated for both the overall cell landscape (Figure 1A) and the tumor-derived cell subpopulations (Figure 1C), together with their expression distribution visualized on the UMAP. In addition, key marker genes were further visualized using bubble plots and UMAP plots to illustrate their expression patterns and distribution across different cell clusters (Figure 1D,E). To distinguish malignant (tumor) cells from non-malignant epithelial cells, single-cell transcriptomic profiles from six normal breast samples in GSE161529 were selected as the reference dataset. Cell annotation using the SingleR algorithm identified 10 major cell types, including T cells, tissue stem cells, endothelial cells, fibroblasts, natural killer (NK) cells, epithelial cells, monocytes, macrophages, neutrophils, and B cells. Epithelial cells were subsequently extracted from the tumor samples for further reclustering (Figure 1B). Using epithelial cells from normal breast samples as the reference, tumor cell subpopulations were then identified with the inferCNV R package (Figure 2A).

### 2.2. Identification of Fatty Acid Metabolism-Related Tumor Cell Clusters in Breast Cancer

To further characterize tumor cell subpopulations associated with fatty acid metabolic programs, we first extracted the transcriptomic profiles of the identified tumor cells and performed consensus clustering using the NMF algorithm implemented in the NMF package. According to the results of cluster analysis, tumor cells were further divided into six distinct subgroups, named Tumor C1–Tumor C6 (Figure 2B). Subsequently, the DoHeatmap function in Seurat was used to visualize the expression mode of the classical marker gene of each tumor subgroup so as to verify differences in molecular characteristics and biological properties of these subgroups (Figure 2C). In order to further analyze the functional characteristics of different tumor subgroups, we downloaded the Hallmark gene set from the MSigDB database (h.all.v2024.1; Hs.symbols), and the differences in pathway activity among the six tumor subgroups were compared. Hallmark pathway enrichment analysis results showed that Tumor C2 shows a high level of enrichment in multiple lipid metabolism-related pathways, including fatty acid metabolism, lipogenesis, cholesterol homeostasis, and peroxisome-related pathways. At the same time, Gene Ontology (GO) analysis further showed that Tumor C2 is also significantly enriched in a variety of biological processes related to fatty acid metabolism, such as fatty acid β-oxidation, lipid oxidation, fatty acid transport, long-chain fatty acid transport, and lipid metabolism regulation, among others (Figure 2D,E). The above results suggest that compared with other tumor subgroups, Tumor C2 has more obvious lipid metabolism reprogramming characteristics, which may provide the energy and biosynthetic substrates required for tumor growth by enhancing fatty acid uptake, transport, and β-oxidation.

### 2.3. Trajectory Analysis of Fatty Acid Metabolism-Related Cell Subpopulations

**Figure 3:**
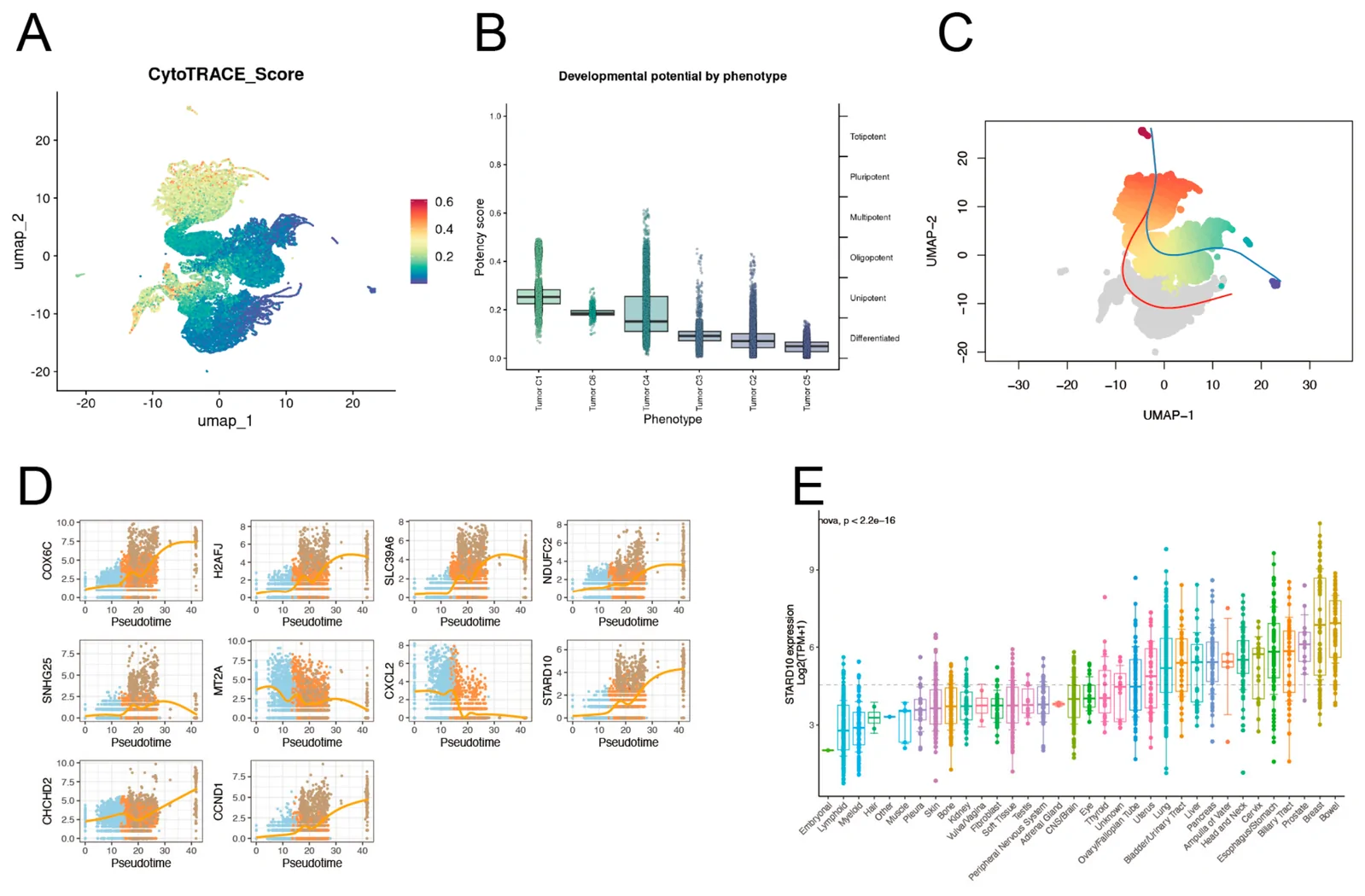
Pseudotime trajectory analysis of tumor cell differentiation. (**A**) UMAP showing the CytoTRACE scores of tumor cells. (**B**) Boxplot showing the CytoTRACE scores of tumor cells. (**C**) Differentiation trajectories of tumor cell subpopulations. (**D**) Dynamic changes in the expression levels of key genes during differentiation. (**E**) Pan-cancer expression level of STARD10. Created in Adobe Illustrator 2023 (version 27.0).

In order to further explore the differentiation status of six tumor cell subgroups and their potential developmental links during the progression of breast cancer, we used the CytoTRACE algorithm to evaluate the differentiation potential of tumor cells and visualize their developmental status. CytoTRACE infers the degree of cell differentiation by integrating gene expression characteristics, which reflects the relative position of cells along the developmental trajectory (Figure 3A). The results showed that there was a significant difference in differentiation potential between the six tumor cell subgroups. The analysis of the box plot showed that the differentiation potential of each subgroup decreased progressively in the order of Tumor C1, C6, C4, C3, C2, and C5, suggesting that the degree of differentiation of Tumor C1 cells is relatively low, while Tumor C5 showed a higher level of differentiation (Figure 3B). It is worth noting that Tumor C2, which was significantly related to fatty acid metabolism, was located at the terminal region of the differentiation trajectory, indicating that this subgroup may represent a specific end-state in the differentiation of breast cancer cells. Given its lowest differentiation potential, Tumor C1 was designated as the putative origin of the differentiation trajectory, and further used the Slingshot package to conduct pseudotime trajectory analysis of tumor cells to characterize the dynamic trajectory of tumor cell development (Figure 3C). The results of pseudotime analysis showed a continuous differentiation trajectory that connected the different tumor cell subgroups, reflecting the significant developmental heterogeneity of breast cancer cells during tumor progression. Subsequently, we conducted dynamic expression analysis of key regulatory genes along the developmental trajectory and screened out ten genes with the most significant changes in the process of pseudotime (Figure 3D). These genes show different time-dependent expression patterns in the process of tumor cell differentiation. Among them, the expression of STARD10 gradually increased with the pseudotime process, and there was a significant upregulation in the later stage of tumor cell differentiation. Based on the results of the above functional enrichment analysis, we speculate that the high expression of STARD10 in fatty acid metabolism-related tumor subgroups (Tumor C2) may participate in lipid metabolism reprogramming and play an important role in the differentiation and progression of breast cancer cells.

### 2.4. Expression Profile and Functional Analysis of STARD10 in Breast Cancer

**Figure 4:**
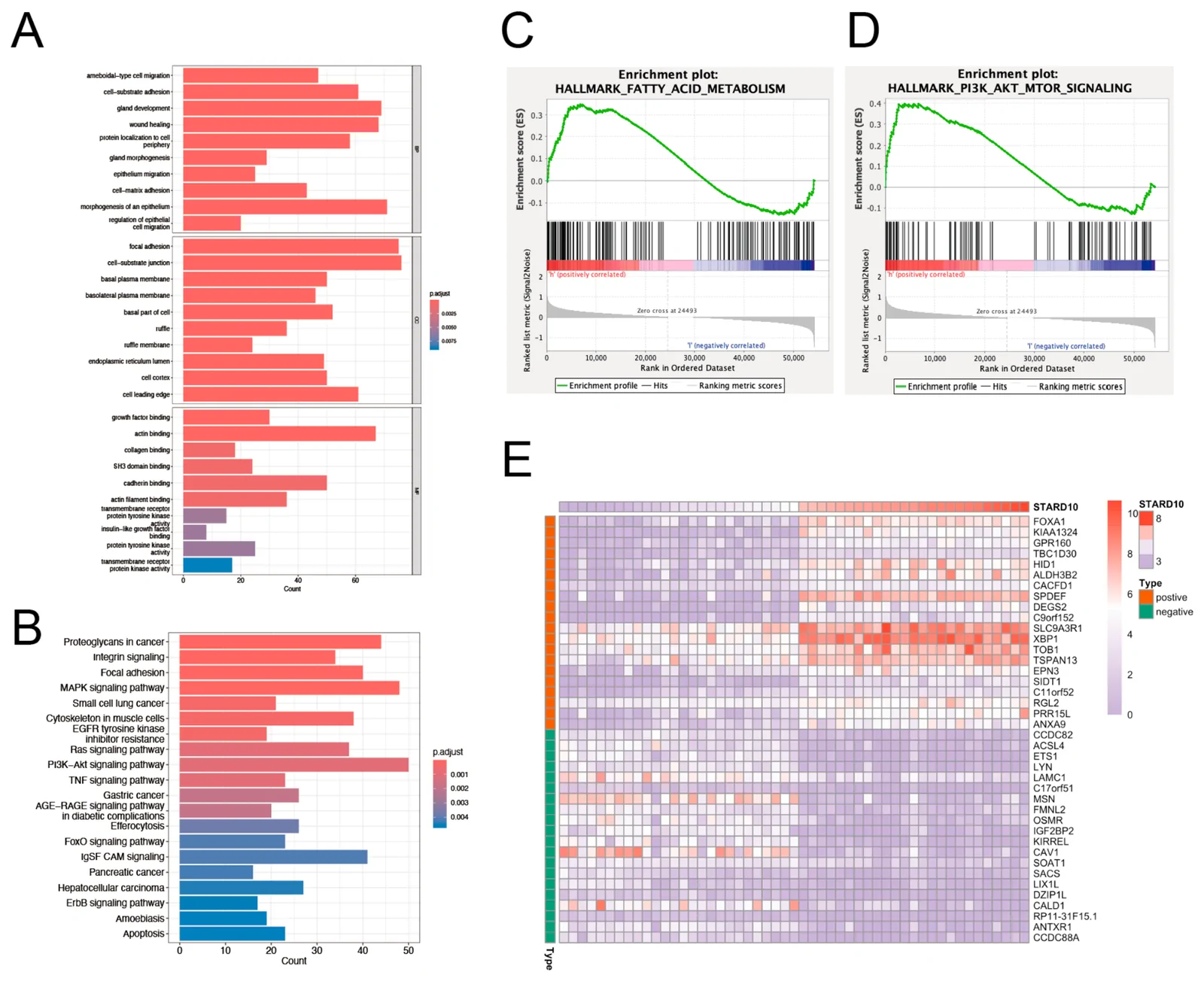
Functional enrichment and GSEA of STARD10-associated genes. (**A**) GO enrichment analysis showing the potential biological functions of STARD10 co-expressed genes in terms of Biological Process (BP), Cellular Component (CC), and Molecular Function (MF). (**B**) KEGG pathway enrichment analysis showing the major signaling pathways and biological processes associated with STARD10 co-expressed genes. (**C**,**D**) Gene Set Enrichment Analysis (GSEA) showing the enrichment of HALLMARK_FATTY_ACID_METABOLISM and HALLMARK_PI3K_AKT_MTOR_SIGNALING among STARD10-related genes. (**E**) Heatmap of STARD10 co-expressed genes, showing the representative genes significantly positively and negatively correlated with STARD10 expression (*n* = 59 cell lines). Created in Adobe Illustrator 2023 (version 27.0).

In order to clarify the expression characteristics of STARD10 across different cancers, we first analyzed the CCLE database. The results revealed that STARD10 was widely expressed across a variety of tumor cell lines, with relatively high mRNA levels observed in several malignancies, including colorectal cancer, breast cancer, and prostate cancer (Figure 3E). Then, we further extracted breast cancer cell lines from the CCLE dataset and used the limma package to identify genes co-expressed with STARD10. A heatmap was subsequently generated to display the top 20 positively and negatively correlated genes associated with STARD10 (Figure 4E). Functional enrichment analysis was further performed on the identified co-expressed genes. In the GO analysis, the Biological Process (BP) category was mainly enriched in epithelial morphogenesis, gland development, and protein localization to the cell periphery. The Cellular Component (CC) category was primarily enriched in focal adhesions, cell–matrix junctions, and the leading edge of the cell. The Molecular Function (MF) category showed enrichment in protein binding, calmodulin binding, and transmembrane receptor protein tyrosine kinase activity (Figure 4A). KEGG pathway analysis further revealed significant enrichment in proteoglycans in cancer, the MAPK signaling pathway, the PI3K-Akt signaling pathway, and the Ras signaling pathway (Figure 4B). To further investigate the potential role of STARD10 in breast cancer progression, Gene Set Enrichment Analysis (GSEA) was performed by comparing gene expression profiles between the STARD10 high- and low-expression groups. The results showed that HALLMARK_FATTY_ACID_METABOLISM and HALLMARK_PI3K_AKT_MTOR_SIGNALING were significantly enriched in the STARD10 high-expression group, suggesting that STARD10 is closely associated with fatty acid metabolism and may contribute to breast cancer progression through the PI3K-Akt signaling pathway (Figure 4C,D).

### 2.5. Molecular Docking Analysis of STARD10 with Key Proteins in the PI3K/Akt Signaling Pathway

**Figure 5:**
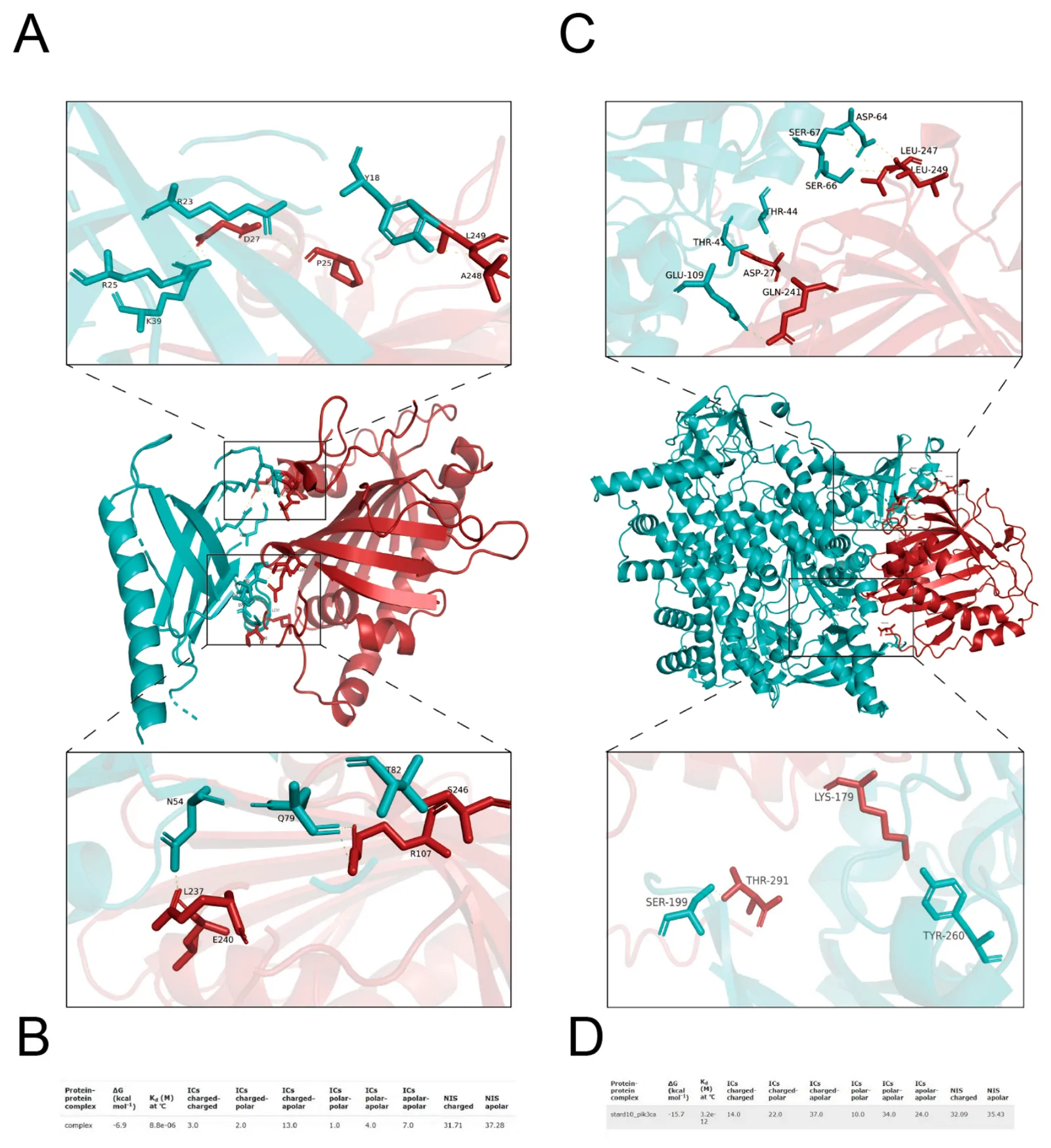
Molecular docking analysis of STARD10 with PI3K/Akt pathway proteins. (**A**) Molecular docking model of STARD10 (red) with AKT1 (cyan) (**B**) Binding energy score of the STARD10-AKT1 complex (−6.9 kcal/mol). (**C**) Molecular docking model of STARD10 (red) with PIK3CA (cyan). (**D**) Binding energy score of the STARD10-PIK3CA complex(−15.7 kcal/mol). Created in Adobe Illustrator 2023 (version 27.0).

The PI3K/Akt signaling pathway is one of the key pathways regulating tumor initiation and progression, participating in diverse biological processes such as cell proliferation, survival, migration, and metabolism. To explore the possible computational link between STARD10 and PI3K/Akt signaling in breast cancer, we performed molecular docking analysis to preliminarily assess the potential binding affinity between STARD10 and the core proteins of this pathway. The docking results showed binding energies of −6.9 kcal/mol between STARD10 and AKT1, and −15.7 kcal/mol between STARD10 and PIK3CA, indicating favorable predicted binding affinities based on computational modeling (Figure 5B,D). The visual analysis of the docking configuration further found that the combination of STARD10 and Akt1 mainly involves the hydrogen bond formed by K39, Y18, Q79, and N54, while its interaction with PI3CA is mainly related to ASP64 and SER66, related to amino acid residues such as SER67 and LYS179 (Figure 5A,C).

### 2.6. Expression and Functional Validation of STARD10 in Breast Cancer

**Figure 6:**
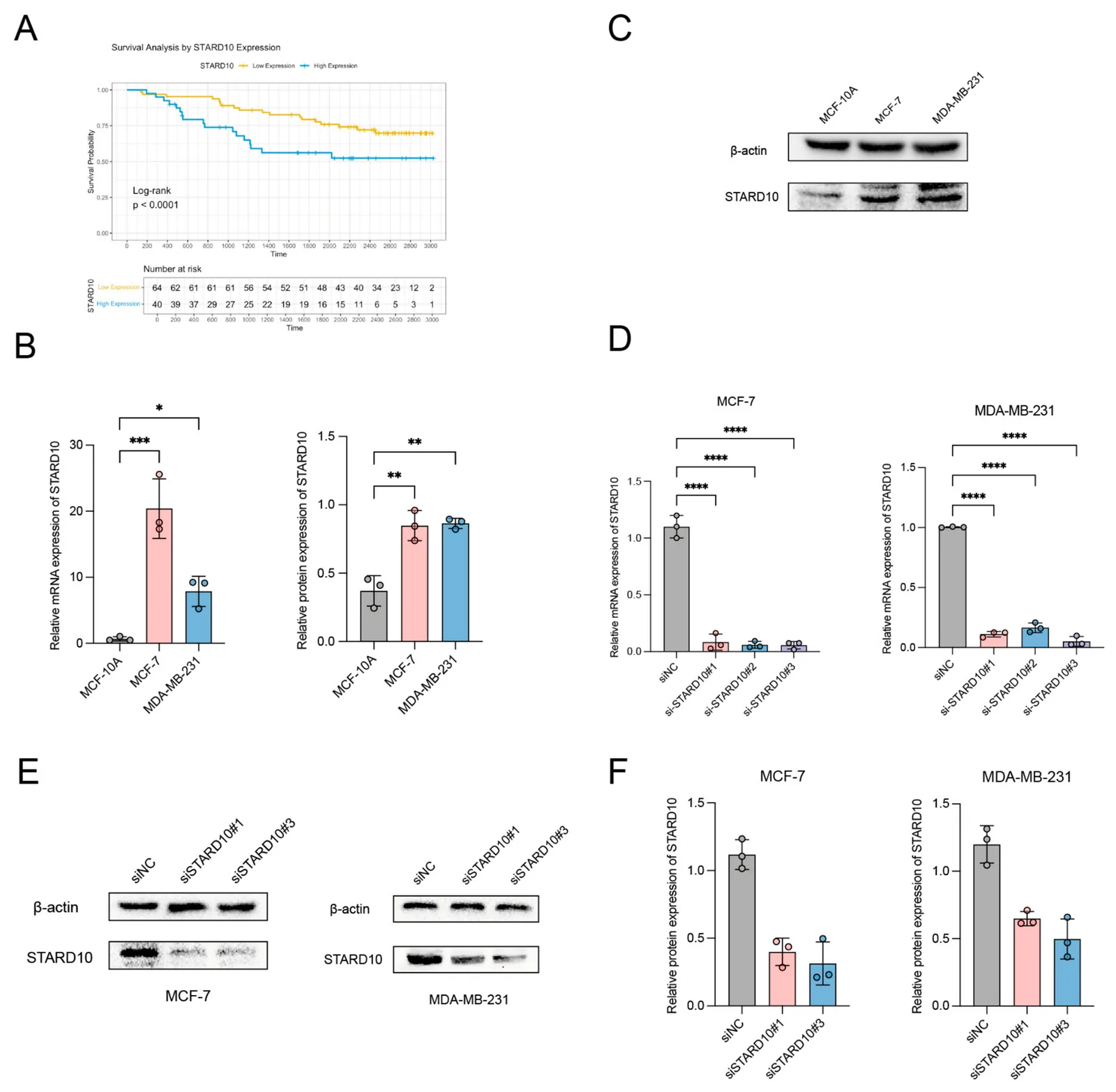
Expression and knockdown efficiency of STARD10 in breast cancer cell lines. (**A**) Analysis of the relationship between STARD10 and OS in breast cancer patients. (**B**,**C**) Western blot and qRT-PCR were used to verify the relative expression of STARD10 between breast cancer cell lines (MCF-7 and MDA-MB-231) and a normal breast epithelial cell line (MCF-10A). (**D**–**F**) MCF-7 and MDA-MB-231 cells were transfected with siSTARD10 or siNC for 48 h, and the expression of STARD10 protein and mRNA was detected by Western blot and qRT-PCR. **** *p* < 0.0001; *** *p* < 0.001; ** *p* < 0.01; * *p* < 0.05.

**Figure 7:**
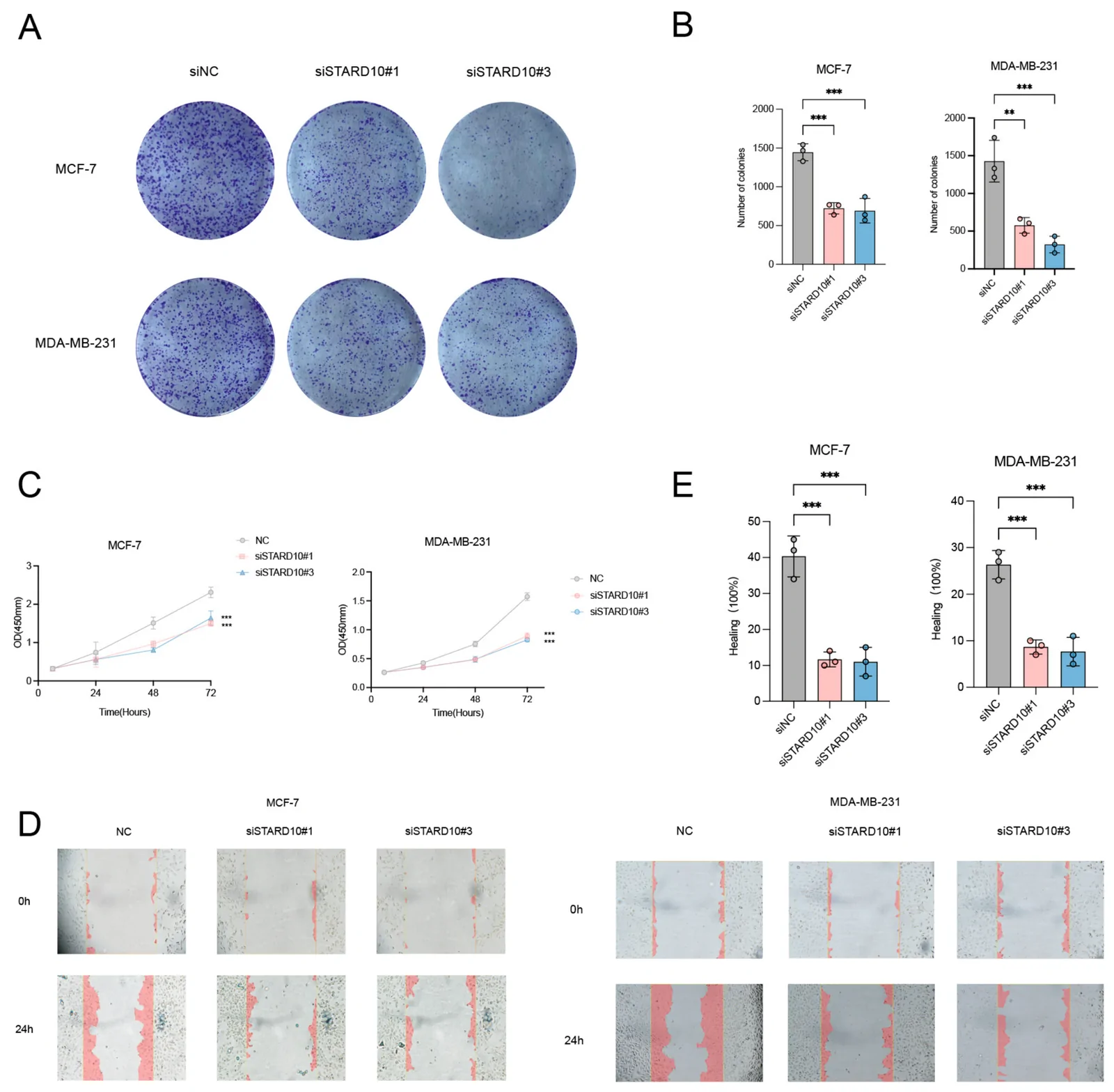
STARD10 knockdown inhibits the proliferation and migration of breast cancer cells in vitro. (**A**,**B**) Colony formation assays were performed in MCF-7 and MDA-MB-231 cells transfected with siNC or siSTARD10. (**C**) CCK-8 assay was used to assess cell viability following siSTARD10 treatment in MCF-7 and MDA-MB-231 cells. (**D**) Wound healing assays were performed in MCF-7 and MDA-MB-231 cells transfected with siNC or siSTARD10. (**E**) Wound healing assays were performed in MCF-7 and MDA-MB-231 cells transfected with siNC or siSTARD10 (bars). ** *p* < 0.01; *** *p* < 0.001.

Survival analysis of 104 breast cancer samples from the GSE42568 dataset revealed that elevated STARD10 expression in tumor tissues was associated with unfavorable clinical outcomes (Figure 6A). To further investigate and validate the biological effects of STARD10 in breast cancer, we measured its expression levels by RT-qPCR. The results showed that STARD10 expression in the breast cancer cell lines MCF-7 and MDA-MB-231 was significantly higher than that in the normal breast epithelial cell line MCF-10A (Figure 6B). Western blot analysis further demonstrated that STARD10 protein expression was also markedly elevated in tumor cells compared with normal breast cells (Figure 6B,C). To further explore the biological role of STARD10 in breast cancer, STARD10 was silenced in MCF-7 and MDA-MB-231 cells using three small interfering RNAs (siRNAs). Transfection efficiency was first verified by RT-qPCR, which showed that all three siRNAs markedly reduced STARD10 mRNA expression (Figure 6D). Based on these results, siSTARD10#1 and siSTARD10#3 were selected for subsequent experiments, and their knockdown efficiency was further confirmed by Western blot. The results showed that both siRNAs significantly reduced STARD10 protein expression in MCF-7 and MDA-MB-231 cells (Figure 6E,F). Cell proliferation was subsequently evaluated in MCF-7 and MDA-MB-231 cells using CCK-8 and colony formation assays. The CCK-8 assay showed that, compared with the siNC group, siSTARD10 significantly inhibited cell proliferation (Figure 7C). Consistently, colony formation assays further confirmed this finding, as the number of colonies formed by MCF-7 and MDA-MB-231 cells was markedly reduced after siSTARD10 transfection (Figure 7A,B). In addition, the effect of STARD10 on breast cancer cell migration was assessed using wound-healing assays, which showed that STARD10 knockdown significantly inhibited the migratory capacity of MCF-7 and MDA-MB-231 breast cancer cells (Figure 7D,E).

### 2.7. STARD10 Regulates PI3K/Akt Signaling and Lipid Metabolism in Breast Cancer

**Figure 8:**
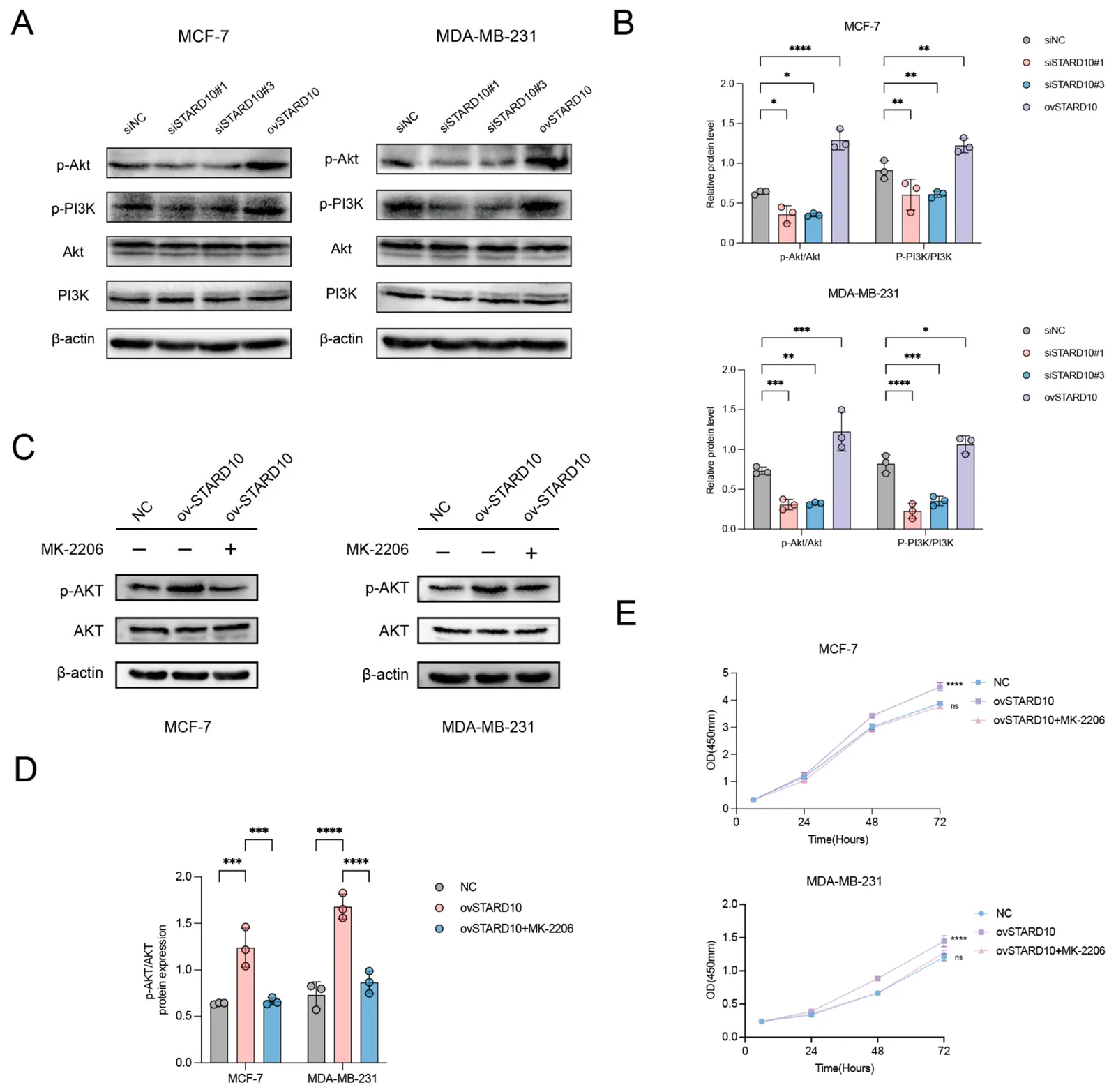
STARD10 regulates PI3K/Akt pathway activation in breast cancer cells. (**A**,**B**) Western blot analysis of p-Akt, p-PI3K, Akt, and PI3K protein expression in MCF-7 and MDA-MB-231 cells. (**C**,**D**) Western blot analysis of p-Akt and Akt protein expression in MCF-7 and MDA-MB-231 cells treated with the Akt inhibitor MK-2206. (**E**) CCK-8 assay showing the proliferation of ov-STARD10 MCF-7 and MDA-MB-231 cells treated with MK-2206. **** *p* < 0.0001; *** *p* < 0.001; ** *p* < 0.01; * *p* < 0.05; ns, not significant.

Gene set enrichment analysis of differential STARD10 expression showed that the PI3K/Akt signaling signature was significantly enriched in breast cancer samples with high STARD10 expression, suggesting a potential link between STARD10 and PI3K/Akt pathway activity. Based on this finding, we hypothesized that STARD10 may promote breast cancer progression through activation of the PI3K/Akt pathway. Consistent with this hypothesis, knockdown of STARD10 markedly reduced the protein levels of p-PI3K and p-Akt in both MCF-7 and MDA-MB-231 cells compared with the si-NC group, whereas STARD10 overexpression increased the expression of these phosphorylated proteins in both cell lines (Figure 8A,B). To further determine whether the increase in p-Akt levels was functionally dependent on Akt activity rather than a nonspecific downstream effect, a rescue experiment was performed using MK-2206, a selective Akt inhibitor. MCF-7 and MDA-MB-231 cells were transfected with the STARD10 overexpression plasmid alone or in combination with MK-2206 treatment. Western blot analysis showed that MK-2206 co-treatment significantly attenuated the increase in p-Akt protein levels induced by STARD10 overexpression in both cell lines (Figure 8C,D). Consistently, CCK-8 assays showed that STARD10 overexpression significantly promoted the proliferation of MCF-7 and MDA-MB-231 cells compared with the NC group, and this proliferative advantage was largely abolished by MK-2206 co-treatment, with no significant difference observed between the ovSTARD10+MK-2206 group and the NC group (Figure 8E). Together, these results demonstrate that STARD10 promotes activation of the PI3K/Akt signaling pathway and cell proliferation in breast cancer cells, and that these effects can be effectively reversed by pharmacological inhibition of Akt, further supporting a functional role for STARD10 in driving PI3K/Akt pathway activity.

**Figure 9:**
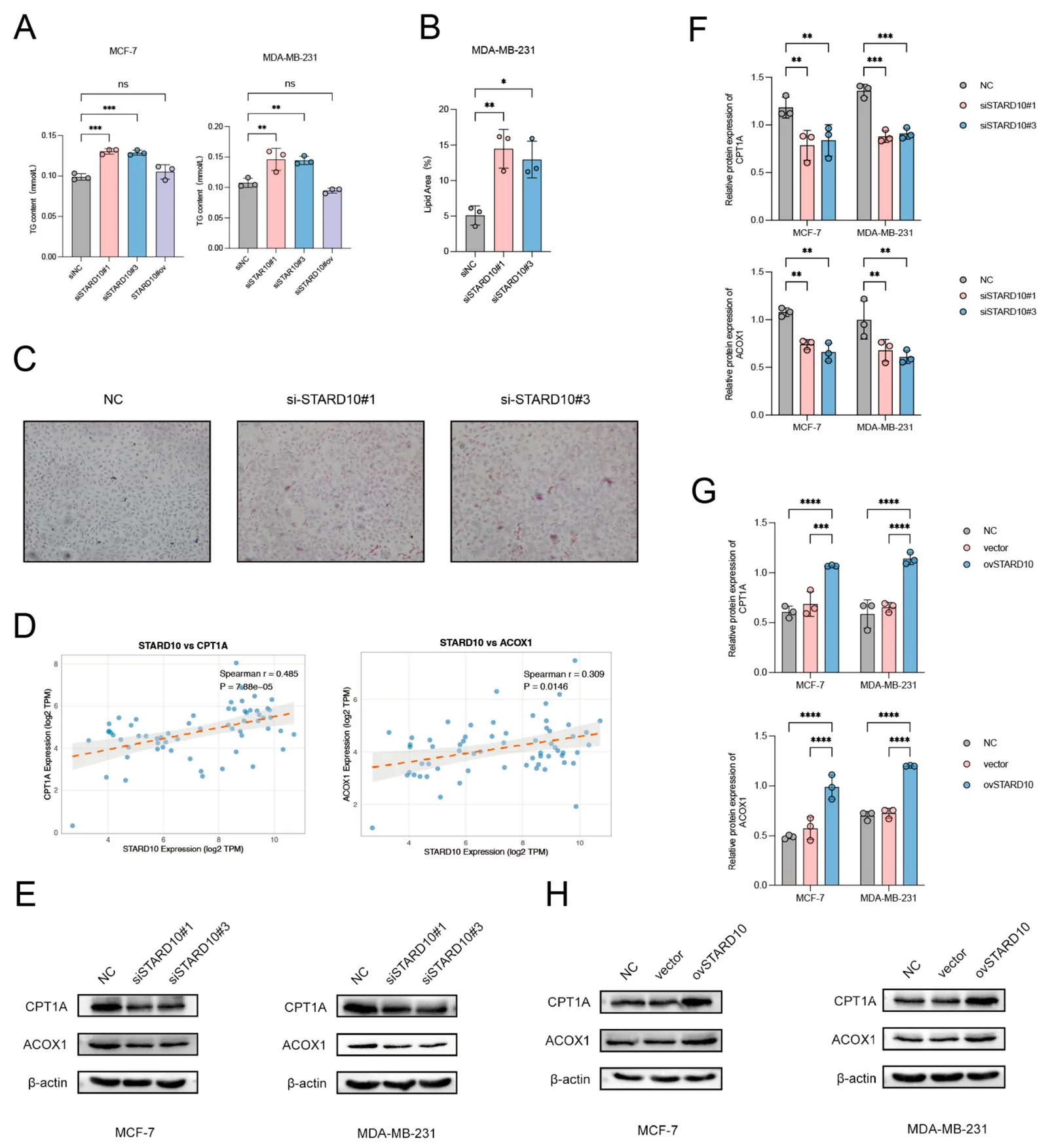
STARD10 regulates fatty acid metabolism in breast cancer cells. (**A**) Intracellular triglyceride (TG) levels in MCF-7 and MDA-MB-231 cells following STARD10 knockdown or overexpression. (**B**,**C**) Oil Red O staining and quantitative analysis showing intracellular lipid droplet accumulation following STARD10 knockdown. Lipid droplet area was quantified using ImageJ software based on color threshold segmentation. (**D**) Correlation analysis between STARD10 and CPT1A (**left**) or ACOX1 (**right**) expression. (**E**,**F**) CPT1A and ACOX1 protein expression following STARD10 knockdown. (**G**,**H**) CPT1A and ACOX1 protein expression following STARD10 overexpression. **** *p* < 0.0001; *** *p* < 0.001; ** *p* < 0.01; * *p* < 0.05; ns, not significant.

Lipid accumulation is often caused by an imbalance among lipid biosynthesis, lipolysis, uptake, and efflux. Previous studies have identified STARD10 as a key regulator of lipid metabolism. Therefore, we further explored its role in lipid metabolic regulation in breast cancer. The results showed that knockdown of STARD10 significantly increased intracellular triglyceride (TG) levels in both MCF-7 and MDA-MB-231 cells, whereas STARD10 overexpression did not cause significant changes in TG content (Figure 9A). This suggests that loss of STARD10 may disrupt intracellular lipid metabolic homeostasis, thereby promoting lipid accumulation. To further verify this observation, we performed Oil Red O staining in MDA-MB-231 cells, which showed a marked increase in intracellular lipid droplet accumulation following STARD10 knockdown, consistent with the TG results (Figure 9B,C). To further elucidate the mechanism underlying STARD10-mediated regulation of lipid metabolism, we examined its correlation with fatty acid β-oxidation-related enzymes using the CCLE database. Correlation analysis revealed that STARD10 expression was significantly and positively correlated with both CPT1A and ACOX1 (Figure 9D), suggesting a potential regulatory relationship between STARD10 and fatty acid β-oxidation. To experimentally validate this relationship, we examined the protein expression of CPT1A and ACOX1 following STARD10 knockdown or overexpression in MCF-7 and MDA-MB-231 cells. Western blot analysis showed that knockdown of STARD10 significantly reduced the protein levels of both CPT1A and ACOX1 in both cell lines (Figure 9E,F), whereas STARD10 overexpression markedly increased the expression of these two enzymes (Figure 9G,H). Collectively, these results demonstrate that STARD10 positively regulates the expression of CPT1A and ACOX1, two key enzymes involved in fatty acid β-oxidation and plays an important role in maintaining lipid metabolic balance in breast cancer cells, further supporting its function as a key regulator of fatty acid metabolism.

## 3. Discussion

Breast cancer has significant molecular heterogeneity and metabolic adaptability, which not only affect the biological behavior of tumor cells, but also further influence the treatment response and clinical outcome. With the rapid development of single-cell transcriptomic technologies, analyzing cell state, developmental evolution trajectory, and metabolic remodeling process from single-cell resolution has gradually become an important means to understand the heterogeneity of breast cancer [14,15,16]. In this study, we integrated multiple single-cell transcriptome data sets of breast cancer and conducted a systematic analysis of tumor cell subgroups. Combined with pseudo-timing analysis, functional enrichment analysis, molecular docking, and in vitro function experiments, we identified a tumor cell subgroup closely related to fatty acid metabolism and revealed that STARD10 may have a tumor-promoting effect in breast cancer.

In a subsequent analysis, we reclassified tumor cells into 6 different subgroups. Among them, Tumor C2 showed significant enrichment in multiple metabolism-related pathways, including fatty acid metabolism, cholesterol homeostasis, peroxidase-related pathways, and fatty acid β-oxidation, suggesting that this subgroup has prominent lipid metabolism reprogramming characteristics. Given that breast cancer occurs in a special microenvironment rich in adipose tissue, fatty acids are not only important raw materials for membrane structure synthesis and energy generation, but also participate in the regulation of oxidative stress, signal transduction, and the shaping of the tumor immune microenvironment. Therefore, the discovery of Tumor C2 further suggests that there is obvious metabolic heterogeneity in breast cancer and indicates that fatty acid metabolism remodeling may play a key role in maintaining certain specific malignant cell states.

STARD10 (StAR-related lipid transfer domain containing 10) belongs to the START domain lipid transport protein family, which is known to be involved in the binding and transport of intracellular lipids. Previous studies have shown that STARD10 has abnormal expression in some breast cancer tissues and cell lines, and has a functional correlation with ErbB/HER2 receptor signaling, which suggests that it may be involved in the maintenance of malignant phenotypes in breast cancer cells [17,18]. Further structural and functional studies have shown that STARD10 can not only bind and transport phosphatidylcholine (PC) and phosphatidylethanolamine (PE), but its lipid-binding pocket may also accommodate phosphatidylinositol molecules. These results suggest that the role of STARD10 is not limited to maintaining the stability of membrane lipid composition, but may also participate in lipid signaling and a variety of biological processes related to cell membranes [19,20]. Overall, STARD10 is likely to play an important role in tumor lipid metabolism remodeling and carcinogenic signal regulation.

In the context of breast cancer, previous studies have reported a functional association between STARD10 and ERBB2/HER2 signaling. Vona-Davis and other studies found that ethanol stimulation can lead to the joint upregulation of STARD10 and ERBB2, and further enhance the proliferation, migration, and invasion ability of breast cancer cells, suggesting that STARD10 may interact with HER2-related signal networks. It is used to promote tumor progression [20]. In addition, the study of Murphy et al. pointed out that in some breast cancer patients, reduced STARD10 expression is associated with poor prognosis [21]. Given that HER2/ERBB2 is an important upstream activation factor recognized by the PI3K/AKT pathway in breast cancer, and the PI3K/AKT axis plays a central role in tumor cell proliferation, survival, invasion and treatment tolerance, therefore, STARD10 and HER2 The connection between the relevant signals suggests that it may further affect the activity of the PI3K/AKT pathway [20,22,23]. However, there is still a lack of clear research evidence on whether STARD10 promotes the reprogramming and malignant progression of breast cancer fatty acid metabolism through the PI3K/AKT pathway.

The PI3K/AKT/mTOR (PAM) signaling pathway is one of the highly conservative and functional signal transduction networks in eucaryotic cells. Under the action of exogenous growth factors, nutrient supply, and other stimulating factors, this pathway can participate in regulating a variety of life activities such as cell survival, proliferation, growth, and metabolism [24,25,26]. Its core composition mainly includes phosphatidyl inositol 3-kinase (PI3K) and serine/threonine protein kinase AKT. A large number of studies have confirmed that abnormal activation of the PAM pathway is widely present in a variety of malignant tumors, about 50% of human cancers, making it one of the most common abnormal carcinogenic signal networks in tumors [27,28,29,30]. For this reason, the continuous over-activation of this pathway has been regarded as an important molecular basis for promoting tumor development, progression, and therapeutic resistance. The activation of the PAM pathway can be caused by a variety of mechanisms, including continuous stimulation of receptor tyrosine kinase (RTKs) or G protein-coupled receptor (GPCRs), abnormal induction of upstream oncogenes, mutation or amplification of key kinases such as PIK3CA, deletion of tumor suppressor gene PTEN, inactivation, and the amplification or functional acquisition mutation of AKT itself, etc. [30,31,32]. In addition, over-activation of the PAM pathway can also promote the occurrence of epithelial–interstitial transformation by regulating cytoskeletal remodeling, cell adhesion, and migration-related processes [33]. In the PI3K family, Class I PI3K is the type that exerts the main lipid kinase activity after responding to the stimulation of growth factors [34]. This kind of enzyme usually exists in the form of a heterodimer, which is composed of the regulatory subunit p85 and the catalytic subunit p110. In terms of mechanism, p85 can use its Src homology 2 (SH2) domain to identify phosphorylated tyrosine residues on activated RTKs, then recruit and activate p110 catalytic subunits to form a biologically active PI3K complex [35]. It is worth noting that PIK3CA, which encodes PI3K p110α catalytic subunit, is one of the most common driving gene changes in human tumors and plays an important role in the course of tumor development [36]. As a key downstream effect molecule in the PAM pathway, AKT also plays a significant role in the occurrence and progression of breast cancer. Previous studies have found that AKT activation has been detected in some pre-breast cancer lesions. In ductal carcinoma in situ and invasive breast cancer, phosphorylated AKT (p-AKT) is often expressed at high levels, and most of the tumors are estrogen receptor positive [37]. In addition, the phosphorylation of AKT at the Ser473 site has been confirmed to be associated with breast cancer metastasis, and about 40% of breast cancers have elevated AKT1 activity [38,39]. In addition to the increase in expression and activity, genetic changes in AKT itself are also an important reason for its abnormal activation. It has been reported that genes encoding different AKT subtypes can undergo amplification and functional acquisition of missense mutations. These changes will further enhance AKT signal output and regulate cell survival, proliferation, growth, apoptosis, glucose metabolism, and other tumor-related biological processes [31,40]. Among them, the most representative molecular event is the E17K point mutation in the AKT1 pleckstrin homology (PH) domain, that is, the 17th position of glutamic acid is replaced by lysine. This mutation can promote the continuous placement of AKT1 in the plasmic membrane, thus enhancing its continuous activation level and leading to increased p-AKT expression in tumor cells [41]. Further studies have shown that p-Akt is overexpressed in 33% of ductal carcinoma in situ (DCIS) cases and 38% of invasive breast cancers, with the majority of these cases (79%) being estrogen receptor-positive, suggesting that aberrant activation of Akt signaling may be involved in the early development of breast tumors and disease progression, and may be associated with hormone receptor status and related biological behaviors [38,39].

This study has several limitations. First, it should be emphasized that the interaction results between STARD10 and AKT1/PIK3CA rely solely on in silico docking predictions and cannot serve as direct proof of physical protein–protein binding. Follow-up experiments, including co-immunoprecipitation, proximity ligation assay, or surface plasmon resonance, are needed to verify the direct physical interaction between STARD10 and AKT1 as well as PIK3CA. Nonetheless, combined with KEGG and GSEA enrichment data, these computational analyses offer preliminary evidence to raise a scientific hypothesis, which supports further mechanistic exploration of the potential functional crosstalk between STARD10 and PI3K/Akt signaling during breast cancer progression. Second, although we demonstrated STARD10-mediated changes in lipid accumulation and the expression of key fatty acid β-oxidation enzymes (CPT1A and ACOX1), direct functional assays measuring fatty acid oxidation flux, such as Seahorse XF analysis, and fatty acid uptake assays were not performed in this study. Future studies incorporating these functional metabolic assays will be necessary to more definitively confirm the fatty acid metabolic phenotype associated with STARD10 in breast cancer cells. Third, among the three integrated single-cell datasets used in this study, only GSE176078 provided molecular subtype annotations at the patient level, precluding a systematic characterization of the molecular subtype distribution of the fatty acid metabolism-associated Tumor C2 subgroup across the full integrated cohort. Future studies incorporating datasets with complete molecular subtype annotations will be needed to further resolve the clinical and molecular subtype identity of Tumor C2 across breast cancer subtypes such as luminal A/B, HER2-enriched, and triple-negative disease.

## 4. Materials and Methods

### 4.1. Data Sources and Preprocessing

The data of single-cell RNA sequencing (scRNA-seq) for breast cancer used by this research institute comes from the Gene Expression Omnibus (GEO) database. The study included a total of three breast cancer data sets, namely GSE180878, GSE176078, and GSE195861, and selected 6 normal breast samples in GSE161529 as controls. First, import the original counting matrix into the R software (version 4.3.2) and build the Seurat object through the CreateSeuratObject function in the Seurat package. Subsequently, quality control was carried out, cells with a ratio of mitochondrial transcripts exceeding 20% were removed, and cells with detected genes between 200 and 7000 were retained; at the same time, genes expressed in fewer than 3 cells were removed. After that, use the NormalizeData function to standardize the data. 5000 highly variable genes were further screened through the FindVariableFeatures function, and principal component analysis (PCA) was carried out using the RunPCA function based on these genes. The number of principal components is evaluated by the ElbowPlot function, and finally, the first 50 principal components are selected for follow-up analysis. Finally, use the harmony R package to complete batch effect correction and data integration.

### 4.2. Gene Set Enrichment Analysis

This study uses gene set enrichment analysis (GSEA) to evaluate whether there is significant enrichment of preset gene sets between different biological conditions or phenotypic groups. The characteristic gene sets required for analysis come from the Molecular Signatures Database (MSigDB). The specific enrichment analysis is completed through the SingleSeqGset R package (version 4.0.5), and the corrected *p*-value is less than 0.05 as the statistically significant criterion for determining the difference. Finally, representative pathways are selected from the analysis results for subsequent visual display.

### 4.3. Analysis of the CCLE Database

The Encyclopedia of Cancer Cell Lines (CCLE) is a relatively systematic public database that integrates molecular characteristics of a variety of tumor cell lines, covering multiple levels such as transcriptome, genome, and drug response. This study obtained transcriptome data from the CCLE database for systematic evaluation of the expression characteristics of STARD10 in different tumor cell lines. Then, breast cancer cell line samples were further screened, and co-expression analysis related to STARD10 was carried out with the help of the limma package in the R language. After calculating the correlation based on the expression level of STARD10, the genes significantly related to it were screened out. Finally, use the pheatmap package to visualize the first 20 genes that are positively correlated and negatively correlated with STARD10, and draw a co-expression pattern heat map.

### 4.4. CytoTRACE Analysis

This study uses CytoTRACE to infer the differentiation status of tumor cells in single-cell RNA sequencing data. Generally speaking, cells with a lower degree of differentiation usually have higher transcription diversity, while cells with a higher degree of differentiation show relatively low gene expression complexity. First, extract the expression matrix from the annotated tumor cells and use CytoTRACE2 (version 1.1.0) to calculate the CytoTRACE score of each cell. A higher CytoTRACE score usually indicates a lower degree of cell differentiation and stronger developmental potential. Subsequently, the CytoTRACE score was summarized according to the tumor cell subgroup, and differences in differentiation potential between different subgroups were shown through the box line diagram.

### 4.5. Slingshot Trajectory Analysis

This study adopts the Slingshot method to reconstruct the cell differentiation trajectory on the basis of integrating dimension-reduction results and cell clustering information. During the analysis process, the pre-annotated cell subgroup is used as the input data, and the pseudo-chronological analysis is carried out with the help of the Slingshot R package (version 2.14.0). Subsequently, the genes with significant dynamic changes along the inference trajectory were further screened out, and the trend of their expression changes was visualized.

### 4.6. Molecular Docking

The AKT1 and PI3CA three-dimensional structures used by this research institute come from the Protein Data Bank (PDB) database. Before carrying out molecular docking, the protein structure should be pretreated, including removing water molecules, adding hydrogen atoms, and optimizing protein conformation. Then, AutoDock Vina (version 1.2.4) was used for molecular docking analysis to evaluate the possible binding method and binding affinity between STARD10, AKT1, and PI3CA. Calculate the binding energy for different docking configurations, and define the complex with the lowest binding energy as the optimal combination model. Finally, the docking results are visualized with the help of PyMOL (version 3.1) to show the potential binding interface and molecular interaction.

### 4.7. Cell Line Culture and Transfection

The human normal breast epithelial cell line MCF-10A and the human breast cancer cell lines MCF-7 and MDA-MB-231 used by the research institute were purchased from Wuhan Punosai Life Technology Co., Ltd. (Wuhan, China). Cell culture uses DMEM culture medium containing 10% fetal bovine serum (Biological Industries, Cromwell, CT, USA) and 1% penicillin/streptomycin (Liji, Shanghai, China), and is incubated at 37 °C, 5% CO_2_, and cultured in a constant-temperature humid incubator. A previously synthesized small interfering RNA (siRNA) targeting the STARD10 gene was purchased from GenePharma Inc. (Shanghai, China). Cell transfection was performed using Lipofectamine 3000 (Thermo Fisher Scientific, Waltham, MA, USA) according to the manufacturer’s instructions.

### 4.8. Western Blot

Protein extraction was performed in MCF-7 and MDA-MB-231 cells using a total protein extraction reagent (KGI Biotechnology Ltd., Claremont, CA, USA). Protein concentration was determined using the BCA assay (KGI Biotech Ltd., HongKong, China). Equal amounts of protein were separated on 12% sodium dodecyl sulfate-polyacrylamide gels (SDS-PAGE; Solarbio Technology Co., Ltd., Shenzhen, China) and then transferred onto PVDF membranes (Millipore, Burlington, MA, USA). After the membrane is completed, first place the membrane in 5% skimmed milk powder at room temperature and close it for 1 h, then add the first antibody diluted to 1:5000 and incubate overnight at 4 °C. The primary antibodies used were as follows: anti-STARD10 (mouse, 1:5000, Cat. No. Ag10932, Proteintech, Rosemont, IL, USA), anti-β-actin (rabbit, 1:5000, Cat. No. 66009-1-Ig, Proteintech, IL, USA), anti-p-Akt (rabbit, 1:2000, Cat. No. 66444-1-Ig, Proteintech, IL, USA), anti-Akt (rabbit, 1:5000, Cat. No. 10176-2-AP, Proteintech, IL, USA), anti-p-PI3K (rabbit, 1:1000, Cat. No. AF3241, Affinity Biosciences Ltd., Changzhou, China), anti-PI3K (rabbit, 1:2000, Cat. No. AF6241, Affinity Biosciences Ltd., Changzhou, China), anti-CPT1A (rabbit, 1:5000, Cat. No. 15184-1-AP, Proteintech, IL, USA), and anti-ACOX1 (rabbit, 1:2000, Cat. No. 10957-1-AP, Proteintech, IL, USA). After washing, the membranes were incubated with secondary antibodies (mouse/rabbit, 1:5000, SA00001-1/SA00001-2, Proteintech, IL, USA) at a dilution of 1:10,000 for 1 h at room temperature. The membranes were then exposed to enhanced chemiluminescence (ECL) reagent (Epizyme Biomedical Technology Co., Ltd., Shanghai, China) for 30 s, and protein bands were detected using the Molecular Imager ChemiDoc XRS system (Bio-Rad, Hercules, CA, USA). Western blot bands were subsequently quantified using ImageJ software (Java 1.8.0_345, 64-bit).

### 4.9. Quantitative Real-Time PCR

Total RNA was isolated from the cell lines using the AG RNAex Pro RNA Kit (AG21101, Accurate Biotechnology, Changsha, Hunan, China). Complementary DNA (cDNA) was then synthesized by reverse transcription using the Evo M-MLV RT Kit with gDNA Clean for qRT-PCR (AG11705, Accurate Biotechnology, Changsha, Hunan, China). Quantitative real-time PCR (qRT-PCR) was performed using the SYBR Green Pro Taq HS Premixed qPCR Kit (AG11701, Accurate Biotechnology, Changsha, Hunan, China). Relative gene expression levels were determined using the 2^−ΔΔCt^ method. All data are expressed as the mean ± SD from three independent experiments. The primer sequences were as follows: STARD10: 5′-CAAGAGCTGCGTCATCACCT-3′ (forward), 5′-TGGGAGCCAGGAACTGAGAA-3′ (reverse), β-actin: 5′-CCTGGCACCCAGCACAAT-3′ (forward), 5′-GGGCCGGACTCGTCATACT-3′ (reverse).

### 4.10. CCK-8 Experiment

Cell proliferation was detected by CCK-8 assay. The cells in each group were seeded in 96-well plates at a concentration of 2 × 10^3^ cells/well, attached for 4 h, and incubated for 24, 48, or 72 h, respectively. The medium was discarded at 0, 24, 48, and 72 h, and the CCK-8 detection reagent was added.

### 4.11. Cell Colony Formation Assay

Cells from each group were plated in 3.5 cm culture dishes at a density of 1000 cells per dish. After incubation for 2 weeks at 37 °C with 5% CO_2_, the cells were washed with PBS, fixed in 4% paraformaldehyde for 15 min, and stained with 0.1% crystal violet for 30 min. Colony formation was then assessed under a light microscope or quantified using ImageJ software.

### 4.12. Healing Experiment

Seven hours after transfection, cells from each group were harvested and seeded into six-well plates at a density of 4.0 × 10^5^ cells/mL, with 2 mL of cell suspension added to each well. After the cells are attached to the wall and evenly distributed for 24 h, use a 200 μL gun head to draw 3 parallel scratches in each hole. Then gently wash the cells with PBS 3 times to remove the shed cells, and then add 2 mL of complete culture medium to each hole. And capture scratch images under the inverted microscope at 0 h and 24 h, respectively. The wound width was quantified using ImageJ software (Java 1.8.0_345, 64-bit). Data are expressed as the mean ± SD from three independent experiments.

### 4.13. Oil Red O Staining

For quantification of Oil Red O staining, images were captured from 3 randomly selected fields per group under a light microscope. The stained area corresponding to lipid droplets was quantified using ImageJ software based on color threshold segmentation, and the percentage of lipid-positive area relative to the total field area was calculated. Image acquisition and quantification were performed by an observer blinded to the group allocation to minimize potential bias.

### 4.14. Statistical Analysis

R (version 4.3.2) and GraphPad Prism 9.0 software were used for statistical analysis and graphical visualization of the data. All experiments were reported as the means ± SD. The differences between which were analyzed for significance using Student’s *t*-test for pairwise comparisons or ANOVA for multivariate analysis. **** *p* < 0.0001; *** *p* < 0.001; ** *p* < 0.01; * *p* < 0.05; ns, not significant.

## 5. Conclusions

At present, the relationship between fatty acid metabolism reprogramming and the tumor microenvironment has not been fully clarified, especially in breast cancer; there is still a lack of in-depth research. Our research reveals the important role of STARD10 in the progression of breast cancer and emphasizes the key significance of STARD10 in regulating lipid metabolism homeostasis and PI3K/Akt signaling pathway, while suggesting that there is a complex relationship between fatty acid metabolism-related tumor subgroups and tumor progression. The interaction of the PI3K/Akt signaling pathway can affect the malignant biological behavior of breast cancer cells. We found that STARD10 expression is closely related to fatty acid metabolism activity and PI3K/Akt pathway activation, providing clues to clarify the role of STARD10 in regulating breast cancer metabolism reprogramming. Therefore, our research results suggest that STARD10 may become a potential biomarker and treatment target for breast cancer with clinical application value.

## Data Availability

All the datasets in this study were obtained from public databases, including GSE176078 (https://www.ncbi.nlm.nih.gov/geo/query/acc.cgi?acc=GSE176078; accessed on 16 June 2025), GSE180878 (https://www.ncbi.nlm.nih.gov/gresubmittedeo/query/acc.cgi?acc=GSE180878; accessed on 16 June 2025), GSE195861 (https://www.ncbi.nlm.nih.gov/geo/query/acc.cgi?acc=GSE195861; accessed on 16 June 2025), GSE161529 (https://www.ncbi.nlm.nih.gov/geo/query/acc.cgi?acc=GSE161529; accessed on 16 June 2025).
